# INTRINSIC PACE: PATTERNS OF ASSOCIATION BETWEEN PHYSICAL AND COGNITIVE FUNCTION ACROSS THREE COHORTS

**DOI:** 10.1093/geroni/igae098.0792

**Published:** 2024-12-31

**Authors:** Stephen Kritchevsky, Peggy Cawthon, Eileen Johnson, Terrie Moffitt, Atalie Thompson, Kathy Xie, Michael Miller

**Affiliations:** Wake Forest University School of Medicine, Wake Forest University School of Medicine, North Carolina, United States; California Pacific Medical Center, San Francisco, California, United States; Sutter Health, San Francisco, California, United States; Duke University, Durham, North Carolina, United States; Wake Forest University School of Medicine, Winston-Salem, North Carolina, United States; Duke University, Durham, North Carolina, United States; Wake Forest University School of Medicine, Winston-Salem, North Carolina, United States

## Abstract

Physical and cognitive function are fundamental aging concepts. However, our understanding of their connection is limited by past work using few assessments or limited age ranges. We used canonical correlation to study the structure of associations between large sets of physical and cognitive assessments in two studies of adults age ≥70; the B-NET (Brain Networks and Mobility Function) study (N=192; 6 physical measures and 13 cognitive measures), and SOMMA (the Study of Muscle, Mobility and Aging) (N=879; 9 physical and 6 cognitive measures). In B-NET and SOMMA, the first canonical function explained 26% and 25% of the shared variability between the two measure sets. In both studies, ONLY TIMED assessments (e.g. stair climb time, gait speed (4M and 400M), chair stand pace, digit symbol coding, and trail-making test-B) strongly loaded on the first function. We sought to replicate this finding in the Dunedin Study (N=1037), a cohort of residents born in 1972-73 who completed 6 physical and 12 cognitive measures when participants were 45.We observed that the timed measures had the strongest associations across physical and cognitive domains. For example, WAIS Processing Speed was most strongly correlated with chair stand time (r=.25) and gait velocity over 6M (r=.26). In contrast, untimed physical tests were at best weakly correlated with untimed cognitive tests. For example, grip strength was most highly correlated with WAIS Working Memory, r=.15. Together, these three studies confirm the presence of a previously unidentified construct connecting physical and cognitive function, which we have tentatively named ‘intrinsic pace’.

